# Pathways Contributing to Chemotherapy‐Induced Myotoxicity Are Attenuated by EPA + DHA in a Clinically Relevant Model of Colorectal Cancer

**DOI:** 10.1002/jcsm.70110

**Published:** 2025-11-16

**Authors:** Peter O. Isesele, Bhumi J. Bhatt, Vickie E. Baracos, Sambasivarao Damaraju, Vera C. Mazurak

**Affiliations:** ^1^ Department of Agricultural Food and Nutritional Science University of Alberta Edmonton Alberta Canada; ^2^ Department of Laboratory Medicine and Pathology, Faculty of Medicine and Dentistry University of Alberta Edmonton Alberta Canada; ^3^ Department of Oncology, Faculty of Medicine and Dentistry University of Alberta Edmonton Alberta Canada

**Keywords:** cancer, chemotherapy, immune regulation, omega‐3 fatty acid, RNA sequencing, skeletal muscle

## Abstract

**Background:**

Skeletal muscle loss is a well‐recognized consequence of cancer, and chemotherapy exacerbates myotoxicity through multiple mechanisms. Retaining muscle mass improves tumour response to therapies and tolerance to chemotherapy; hence, interventions to mitigate myotoxicity warrant investigations. This study aimed to investigate the protective effects of dietary eicosapentaenoic acid (EPA) and docosahexaenoic acid (DHA) provided in the form of fish oil on chemotherapy‐induced myotoxicity using next‐generation RNA sequencing (NGS).

**Methods:**

Fischer 344 rats were fed either a standard diet (STD) for the entire study or switched to a diet containing fish oil (2.3 g/100 g of diet) when chemotherapy (irinotecan + 5‐fluorouracil) was initiated. All rats received the Ward colon tumour and tumours were allowed to grow for 2 weeks. Comparisons were made between (1) tumour‐bearing rats on a standard diet (TUMOUR STD), (2) tumour‐bearing rats that received chemotherapy on a standard diet (CHEMO STD) and (3) tumour‐bearing rats that received chemotherapy on a fish oil diet (CHEMO FO). After two cycles of chemotherapy, NGS was performed on gastrocnemius muscle. Differential expression of genes was performed using DEseq2, with a fold‐change cut‐off ≥ 1.5 and *p* value < 0.05. Ingenuity pathway analysis (IPA) was used for functional annotation, canonical pathways and upstream regulators analysis.

**Results:**

Transcriptomic analysis revealed distinct alterations in skeletal muscle gene expression profiles. In the CHEMO STD versus TUMOUR STD comparison, 272 genes showed differential expression. Of these, 55% were upregulated and 45% were downregulated. Two cycles of chemotherapy altered genes in the pathways of proliferation of muscle cells (*p* < 10^−7^), connective tissue disorder (*p* < 10^−6^), apoptosis (*p* < 10^−3^) and neurodevelopmental disorders (*p* < 10^−3^). In the CHEMO FO versus CHEMO STD comparison, 274 genes were differentially expressed (73% upregulated and 27% downregulated). Dietary fish oil exclusively altered immune‐related functions, notably downregulating several genes in the leukocyte extravasation pathway (−log*p* value 5.92, *z* score −2.83). Upstream regulatory molecules after chemotherapy were predicted to inhibit transcription factors involved in myogenic regeneration, while those fed a diet containing fish oil showed inhibition of inflammation‐related cytokines.

**Conclusion:**

Chemotherapy negatively impacts processes involved in muscle homeostasis, including muscle cell proliferation and regeneration. The provision of fish oil primarily showed protective effects from pro‐inflammatory mediators by downregulating genes involved in the leukocyte extravasation pathway. Our findings provide novel insights into the molecular mechanisms underlying chemotherapy‐induced myotoxicity and the potential therapeutic benefits of dietary EPA + DHA to restore muscle homeostasis in cancer.

## 1 Introduction

It is increasingly recognized that drugs used to treat tumours also exert deleterious effects on muscle [1]. Myotoxicity is now considered to be one of the negative effects of systemic treatments impairing muscle function, compromising treatment response and escalating toxicities [2]. Myotoxicity is broadly characterized by disruptions in a litany of pathways including protein turnover, proliferation, differentiation, mitochondrial function and aberrant lipid deposition (reviewed by [3]). Animal models enable a controlled setting by which to gain mechanistic understanding and apply therapeutic strategies to mitigate myotoxicity. Thus far, only a handful of clinical studies have investigated the capacity to reverse the impact of chemotherapy on muscle, and very few mechanistic studies exist [3]. Next‐generation sequencing (NGS) has revolutionized the field of understanding metabolic pathways at the transcript level. While conventional methods are able to quantify a limited number of preselected genes (i.e., PCR), and microarrays offer broader profiling but with inherent limitations in range and the detection of novel transcripts, RNA sequencing (RNA‐seq) enables the high‐throughput sequencing of thousands of RNA molecules in parallel. Advantages over traditional approaches include accurate quantification of both highly and lowly expressed genes, detecting novel transcripts in an untargeted approach, identification of splice variants and unannotated genes, providing a truly comprehensive view of the transcriptome. NGS is a powerful tool that identifies transcriptional changes between two conditions and has been used for studies on tumours [4, 5] but not for muscle exposed to chemotherapy in the tumour‐bearing state. Given that the myotoxicity induced by chemotherapy induces diverse and complex changes in muscle, genome‐wide mRNA profiling is a powerful tool that reveals the molecular intricacies of chemotherapy impact on muscle.

Provision of fish oil, rich in eicosapentaenoic acid (C20:5n‐3; EPA) and docosahexaenoic acid (C22:6n‐3, DHA), has emerged as a potential dietary strategy to counteract chemotherapy's adverse effects on skeletal muscle [6, 7]. In aging and chronic disease, n‐3 fatty acids have been shown to impact mitochondrial function, insulin response and other anabolic pathways [8, 9]. Using a preclinical model of colorectal cancer (CRC), a diet containing fish oil (EPA + DHA) initiated at the start of chemotherapy (irinotecan [CPT‐11] + 5‐fluorouracil [5‐FU]) effectively mitigated chemotherapy‐associated myosteatosis (fat infiltration in muscle) while asserting tumour control [10], which aligns with observations in the clinical setting [11]. Prevention of chemotherapy‐induced myosteatosis observed with provision of fish oil in that study was associated with downregulation of adipogenic transcription factors measured using RT‐qPCR, indicating that EPA + DHA may inhibit fat storage by modulating the expression of genes involved in adipogenesis [10]. Other elements were not evaluated, providing the opportunity herein for an unbiased approach to identifying pathways evoking myotoxicity as a result of chemotherapy and the potential to be modified through a nutritional therapeutic strategy.

Understanding the precise molecular and cellular impact of chemotherapy within muscle in humans is challenged by heterogeneity in patient groups and treatments, adherence to the intervention and the capacity to collect patient biopsies, which is not possible in many oncological settings. This study uses a tumour‐bearing rat model to study chemotherapy's impact on skeletal muscle. This is clinically relevant as chemotherapy is not provided in the healthy condition. We used a chemotherapy regimen that recapitulates therapy for CRC in humans with respect to doses, cycles and toxicity of a combined regimen of CPT‐11 + 5‐FU [12, 13] provided over two cycles. mRNA from muscle was used to assess chemotherapy‐induced transcriptomic changes and the impact of dietary EPA + DHA provided over the same period [10]. Unbiased genome‐wide mRNA profiling was used to explore differentially expressed transcripts, diseases and biological functions, insights into upstream regulators and canonical pathways altered after two cycles of chemotherapy treatment and the effect of dietary EPA + DHA in the understanding of the potential benefits to muscle.

## 2 Materials and Methods

### 2.1 Animal Model and Experimental Design

**FIGURE 1:**
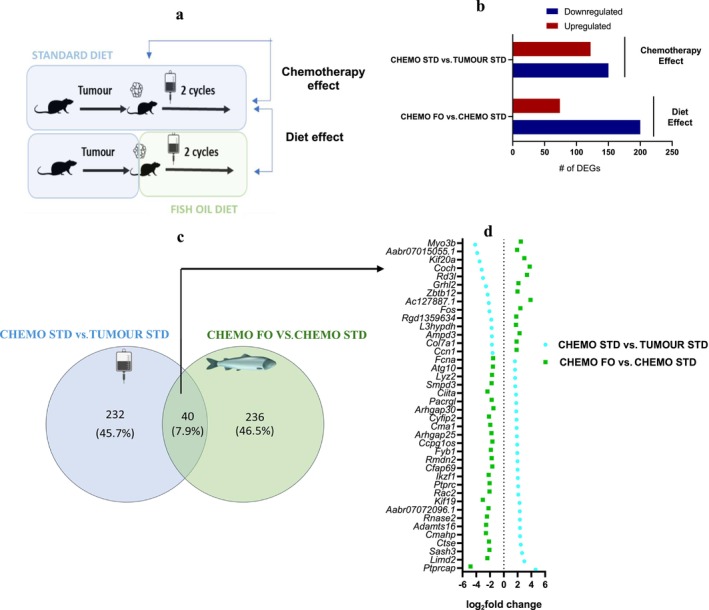
Number of upregulated, downregulated and overlap genes in the assessment of chemotherapy effects and diet effects. (a) Experimental design and differential gene expression comparison groups denoting the chemotherapy effect and the diet effect comparisons, (b) the total number of upregulated and downregulated genes in both comparisons, (c) Venn diagram of the DEGs in both comparisons showing the overlap and (d) comparison of differentially expressed genes showing those altered by chemotherapy on a standard and fish diet (EPA + DHA). CHEMO—chemotherapy, CHEMO FO—tumour‐bearing animals that received chemotherapy, consumed fish oil diet, started the same day as chemotherapy (*n* = 8), CHEMO STD—tumour‐bearing animals that received chemotherapy (*n* = 7), consumed standard diet, FO—fish oil, STD—standard diet, TUMOUR STD—tumour‐bearing animals that did not receive chemotherapy (*n* = 5), consumed standard diet.

The study was approved by the University of Alberta Animal Care and Use Committee and conducted in accordance with the Guidelines of the Canadian Council on Animal Care. The experimental design has previously been described, and the *gastrocnemius* muscles used here were those archived from that study in order to investigate pathways contributing to myotoxicity and those amenable to a dietary intervention [10]. Briefly, 1 week before tumour implantation, female Fischer 344 rats (*n* = 72) weighing an average of 127 ± 18 g aged 11–12 were randomly assigned to one of three treatment arms: tumour‐bearing group on a standard diet (STD) that did not receive chemotherapy (TUMOUR STD [*n* = 8]), tumour‐bearing group that received two cycles of chemotherapy on standard diet during chemotherapy (CHEMO STD [*n* = 8]) and tumour‐bearing group that received two cycles of chemotherapy on diet containing fish oil (CHEMO FO [*n* = 8]; Figure 1a). For the latter group, the standard diet was switched to a diet containing EPA + DHA at the start of chemotherapy. Rats in the CHEMO STD group and CHEMO FO group underwent two cycles of chemotherapy as previously described [10]. The study design and rationale for muscle transcriptomic analyses using NGS on the gastrocnemius muscle biopsies are shown in Figure 1a. This analysis enables understanding of molecular mechanisms to address firstly, the specific effect of repeated cycles of chemotherapy in the tumour‐bearing state (chemotherapy effect) and secondly, the pathways by which dietary EPA + DHA (diet effect) modifies the muscle transcriptome response to chemotherapy.

### 2.2 Tumour Implantation and Chemotherapy Administration

Tumour implantation, chemotherapy administration and accrual of *gastrocnemius* muscle were described previously [10]. Briefly, the Ward colorectal primary carcinoma (0.05 g; supplied by Dr. Y. Rustum, Roswell Park Institute Buffalo, NY, USA) was allowed to grow for 2 weeks (Days −14 to 0). On Day 0 (the first day of chemotherapy), CPT‐11 was administered, while 5‐FU was administered the following day (50 mg/kg bw) representing one cycle of chemotherapy. The same combination (Cycle 2) was given on Days 7 and 8, respectively. Rats were sacrificed and gastrocnemius muscle harvested 7 days later [10].

### 2.3 Diet Composition

The components of both semipurified, isocaloric and isonitrogenous standard and fish oil diets have previously been described [10]. Both diets provided 20% of total calories from protein, 40% from fat and 40% from carbohydrates. Saturated, monounsaturated and polyunsaturated content were similar between the standard and fish oil diets with only the amount of EPA + DHA content (**3.5% w/w)** different between the diets. The amount of EPA and DHA provided aligns with levels consumed by humans in clinical trials [11]. Body weight, food intake and tumour measurement procedures have been previously reported [10].

### 2.4 RNA Extraction

The MagMax‐96 total RNA isolation Kit (Ambion, Austin, TX, USA) was used to extract total RNA from gastrocnemius muscle (10 mg/*n* ≥ 5 per group) according to the manufacturer's protocol. The quantity and quality of extracted RNA were determined using a NanoDrop spectrophotometer (Thermo Scientific, Wilmington, DE) and an Agilent 2100 Bioanalyzer (Agilent Technologies, Santa Clara, CA, USA). RNA integrity number (RIN) from Bioanalyzer were ≥ 9.0, indicating high quality of RNA isolated from muscle biopsies, and these were subjected to NGS analysis.

### 2.5 Next‐Generation RNA‐seq and Bioinformatic Analyses

PlantBiosis Ltd. (Lethbridge, Alberta, Canada) provided NGS services from library preparation and sequencing. The TruSeq Stranded Total RNA with Ribo‐Zero Human/Mouse/Rat kit was used to make RNA‐seq libraries, which were prepared according to the manufacturer's instructions. For rRNA depletion, 1 μg of total RNA per sample was employed as an input, which was further purified, fragmented and used for cDNA synthesis. There were *n* = 5, *n* = 7 and *n* = 8 samples sequenced from TUMOUR STD, CHEMO STD and CHEMO FO, respectively. All samples were sequenced on an Illumina NextSeq 500 with high‐throughput 2 × 150 nt (paired‐end sequencing) runs at a density of 35 samples per flow cell, resulting in 10–13 million reads per sample. Illumina CASAVA 1.9 with default parameters was used for base calling and demultiplexing. Trim Galore v.0.4.1 was used to trim the adapters. FastQC v.0.11.4 was used to check the quality of the sequenced reads. Tophat 2.0.10 and Bowtie2 were used to match the trimmed sequences to the rat reference genome. The rat genome (Rnor 6, Ensembl) was used as a reference genome and was downloaded from the iGENOME website. Sequences that were aligned were saved as .sam files, which were subsequently transformed to .bam files and used for further data processing. Partek Flow software was used to analyse the data in the (.bam) files. Ensembl *Rattus norvegicus* Rnor‐6.0.104 was used to annotate mRNAs. DEseq2 was used to perform DEG analysis (fold‐change cut‐off ≥ 1.5 and *p* value < 0.05) [14]. Data have been deposited in NCBI's Gene Expression Omnibus and are accessible through GEO series accession number GSE281112.

### 2.6 Functional Annotation, Pathway and Statistical Analysis

The DEGs from the transcriptome analysis were subjected to in silico functional annotation using ingenuity pathway analysis (IPA, QIAGEN Inc., https://digitalinsights.qiagen.com/IPA) to identify canonical pathways, diseases and biological functions and upstream regulators. Upstream analysis examines the expected effects between upstream regulators and downstream target genes from the manually curated IPA knowledgebase. If the direction of expression (up or down) of the transcriptional regulator in the study is consistent with those observed from the manually curated results for that target regulator in the IPA database, the predicted state of effect is considered activated (*z* score > 2) or inhibited (*z* score < −2), respectively. IPA calculates a separate *p* value (*p* value of overlap using Fischer's exact test) for biological process prediction. The −log(*p* value) > 1.3 (equivalent to a nominal *p* value of 0.05) was used as a threshold for canonical pathway analysis, diseases and functions. For upstream regulators, the *p* value of overlap < 0.05 was set as the threshold to signify the overlap between the genes in the data set to those by the upstream regulator indicated in IPA. TUMOUR STD was compared with CHEMO STD to determine the chemotherapy effect, while CHEMO STD was compared with CHEMO FO to evaluate the effect of fish oil (Diet effect). Histogram plots were made using GraphPad Prism (Version 8.0, GraphPad Software Inc.).

## 3 Results

### 3.1 Transcriptomic Impact of Chemotherapy: A Comparison of Muscle Transcriptome Between Animals Receiving Two Cycles of Chemotherapy on a Standard Diet and Tumour‐Bearing Animals

To evaluate the chemotherapy effect, a comparative analysis of gene expression profiles between CHEMO STD versus TUMOUR STD (Table 1) was conducted. A total of *N* = 272 genes were found to be differentially expressed in the CHEMO STD versus TUMOUR STD comparison, with 150 genes (55%) upregulated and 122 genes (44%) downregulated (Figure 1b). The full list of the differentially expressed genes is shown in Table S1. The full list of all diseases and biological functions is shown in Table S2. *N* = 58 canonical pathways were identified from the canonical pathway analysis at −log(*p* value) threshold of 1.3 (or *p* value ≤ 0.05; Table S3).

**TABLE 1:** Diseases and functional annotation.

Exclusive to animals receiving chemotherapy on a standard diet (CHEMO STD)	Common to all animals receiving chemotherapy (CHEMO STD and CHEMO FO)	Exclusive to animals receiving chemotherapy on a diet containing EPA + DHA (CHEMO FO)
Abnormal morphology/abnormal development		
Proliferation of muscle cells *p* < 10^−7^		
Development of body trunk *p* < 10^−6^		
Limb defect *p* < 10^−4^		
Connective tissue disorder *p* < 10^−6^		
Differentiation of connective tissue *p* < 10^−4^		
Cell death		
Organismal death *p* < 10^−5^		
Necrosis *p* < 10^−3^		
Apoptosis *p* < 10^−3^		
Metabolic disease		
Glucose metabolism disorder *p* < 10^−5^		
Nervous system		
Migration of neurons *p* < 10^−3^		
Density of neurons *p* < 10^−3^		
Neurodevelopmental disorder *p* < 10^−3^		
	Immune response	
*p* < 10^−7^	Function of leukocytes	*p* < 10^−14^
*p* < 10^−6^	Recruitment of leukocytes	*p* < 10^−4^
*p* < 10^−5^	Leukocyte migration	*p* < 10^−13^
*p* < 10^−5^	Inflammatory response	*p* < 10^−7^
*p* < 10^−3^	Quantity of lymphatic system cells	*p* < 10^−15^
*p* < 10^−4^	Proliferation of blood cells	*p* < 10^−12^
*p* < 10^−4^	Quantity of thymocytes	*p* < 10^−5^
*p* < 10^−4^	Chemotaxis	*p* < 10^−5^
*p* < 10^−4^	Proliferation of immune cells	*p* < 10^−13^
*p* < 10^−4^	Systemic autoimmune syndrome	*p* < 10^−5^
*p* < 10^−4^	Proliferation of lymphocytes	*p* < 10^−13^
*p* < 10^−3^	Quantity of IgG	*p* < 10^−6^
*p* < 10^−3^	Function of antigen‐presenting cells	*p* < 10^−5^
		*p* < 10^−13^ Quantity of T lymphocytes
		*p* < 10^−11^ Activation of T lymphocytes
		*p* < 10^−8^ Apoptosis of mononuclear leukocytes
		*p* < 10^−7^ Quantity of granulocytes
		*p* < 10^−7^ Cell tethering or rolling of leukocytes
		*p* < 10^−11^ Cell movement of lymphocytes
		*p* < 10^−8^ Cell death of immune cells
		*p* < 10^−7^ Quantity of CD4 + T lymphocytes
		*p* < 10^−5^ Cellular infiltration by myeloid cells
		*p* < 10^−5^ Cell rolling of neutrophils
		Nervous system
		*p* < 10^−5^ Abnormal conduction by nerves

*Note:* For the common immune response, the *p* value on the left represents that of the control versus tumour (chemotherapy effect), while the *p* value on the right represents that of the fish oil versus control diet (diet effect) comparison.

Abbreviations: CHEMO—chemotherapy, CHEMO FO—tumour‐bearing animals that received chemotherapy—consumed fish oil diet—started the same day as chemotherapy, CHEMO STD—tumour‐bearing animals that received chemotherapy—consumed control diet, DHA—docosahexaenoic acid, EPA—eicosapentaenoic acid, FO—fish oil, STD—standard diet.

#### 3.1.1 Chemotherapy Altered Immune‐Related Functions and Cell Death Functions

Chemotherapy profoundly influenced immune‐related pathways. Functional annotation revealed altered expression of genes involved in immune cell populations, including lymphatic system cells, thymocytes and blood cells, as well as immunoglobulin G (IgG) levels and antigen‐presenting cell (Table 1). Marked activation of cell death functions, including organismal death, necrosis and apoptosis, was observed (*p* < 10^−3^; Table 1). The gene with the highest *p* value in the differential expression associated with chemotherapy was *Cd209f*, with a log_2_ FC of 8.20 (Table 2). *Cd209f* plays a key role in the induction of immune responses against numerous pathogens by modulating TLR‐induced activation [15]. The expression of genes involved in the complement system, a pivotal bridge between innate and acquired immunity, was increased following chemotherapy. The complement component 3 (*C3*), a central and abundant factor in complement system activation and the classical pathway subcomponents *C1qb* and *C1qc* were all increased (Table S3) in response to chemotherapy in rats on a standard diet. The classical pathway subcomponents *C1qb*, *C1qc* and *C3* mediate inflammation by regulating leukocyte adhesion, and migration exhibited elevated expression levels postchemotherapy.

**TABLE 2:** Chemotherapy effect: Top 15 upregulated and downregulated differentially expressed genes altered by chemotherapy (CHEMO STD vs. TUM STD).

Gene name	Description	log_2_fold change	*p*
Upregulated			
*Cd209f*	CD209f molecule	8.20	5.00E‐04
*Ptprcap*	Protein tyrosine phosphatase, receptor type, C‐associated protein	4.61	8.09E‐03
*Cish*	Cytokine inducible SH2‐containing protein	3.70	2.17E‐03
*Arrdc2*	Arrestin domain containing 2	3.31	1.59E‐02
*Ms4a6bl*	Membrane‐spanning 4‐domains, subfamily A, member 6B‐like 1	3.16	1.82E‐02
*Spata1*	Spermatogenesis associated 1	3.07	7.54E‐03
*Limd2*	LIM domain containing 2	2.98	3.10E‐03
*Gadd45g*	Growth arrest and DNA damage‐inducible gamma	2.96	3.24E‐04
*Adgb*	Androglobin	2.82	4.28E‐02
*Cyb5d2*	Cytochrome b5 domain containing 2	2.79	2.74E‐04
*Ntsr1*	Neurotensin receptor 1	2.79	1.26E‐02
*Frat2*	FRAT regulator of WNT signalling pathway 2	2.77	4.63E‐02
*Hpgd*	15‐Hydroxyprostaglandin dehydrogenase	2.77	1.66E‐02
*Sash3*	SAM and SH3 domain containing 3	2.65	3.81E‐03
*Mnda*	Myeloid cell nuclear differentiation antigen	2.63	3.71E‐05
Downregulated			
*Caskin1*	CASK interacting protein 1	−2.44	2.50E‐03
*Opcml*	Opioid binding protein/cell adhesion molecule‐like	−2.45	1.19E‐02
*Ptx4*	Pentraxin 4	−2.47	1.36E‐02
*Mmp17*	Matrix metallopeptidase 17	−2.49	1.58E‐02
*Pla2g4e*	Phospholipase A2, group IVE	−2.58	2.33E‐02
*Grhl2*	Grainyhead‐like transcription factor 2	−2.59	1.47E‐02
*Ntrk3*	Neurotrophic receptor tyrosine kinase 3	−2.63	3.46E‐03
*Il17re*	Interleukin 17 receptor E	−2.84	2.74E‐02
*Lrrc55*	Leucine‐rich repeat containing 55	−2.93	3.62E‐03
*Rd3l*	RD3 like	−3.03	1.08E‐02
*Lrrc52*	Leucine‐rich repeat containing 52	−3.12	7.03E‐03
*Coch*	Cochlin	−3.22	4.13E‐02
*Chl1*	Cell adhesion molecule L1‐like	−3.51	5.31E‐03
*Kif20a*	Kinesin family member 20A	−3.52	1.29E‐02
*Myo3b*	Myosin IIIB	−4.16	1.58E‐03

*Note:* Tumour‐bearing animals were compared to animals that received chemotherapy; both consumed a standard diet. Positive log_2_fold changes are upregulated genes, while negative log_2_fold changes are downregulated genes. Shaded rows are those genes that are overlapping between the chemotherapy effect and the diet effect ([CHEMO STD vs. TUM STD] and [CHEMO FO vs. CHEMO STD]). *p* value < 0.05 was considered significant.

Abbreviations: CHEMO—chemotherapy, STD—standard diet.

The comparison between CHEMO STD versus TUMOUR STD predicted alterations in upstream regulators that modulate downstream effector genes associated with immune response (Table S4). Several upstream regulators involved in immune modulation were altered by chemotherapy. Interferon gamma (IFNG), a pro‐inflammatory cytokine known to mediate immune activation and muscle inflammation, was predicted to be activated (*z* score = 2.12). Cyclosporin A, an immunomodulatory agent that interferes with calcium signalling and T‐cell activation, was also predicted to be activated (*z* score = 1.83). In contrast, components of the transforming growth factor beta (TGFβ) family, particularly TGFB1 (*z* score = −2.43, *p* value = 5.21E‐08), were predicted to be inhibited, reflecting a suppression of tissue repair and immune‐regulatory pathways. Mothers against decapentaplegic homologue 3 (SMAD3), a key intracellular effector of TGFβ signalling that regulates inflammation and fibrosis, was likewise predicted to be inhibited (*z* score = −2.53, *p* value = 4.61E‐07), further supporting an overall disruption of anti‐inflammatory signalling cascades.

#### 3.1.2 Chemotherapy‐Induced Disruption of Glucose Metabolism and Energy Homeostasis Effects

Metabolic disturbances, particularly glucose metabolism disorders, were also identified in the chemotherapy group (Table 1). Significant disruptions in glucose metabolism were evident, alongside predicted inhibition of regulators implicated in energy balance and muscle homeostasis. Upstream regulator including leptin (LEP) (*z* score = −2.30), which plays a role in energy balance and muscle metabolism was predicted to be inhibited (Table S4).

#### 3.1.3 Chemotherapy‐Induced Structural Disruption of Skeletal Muscle and Extracellular Matrix

**FIGURE 2:**
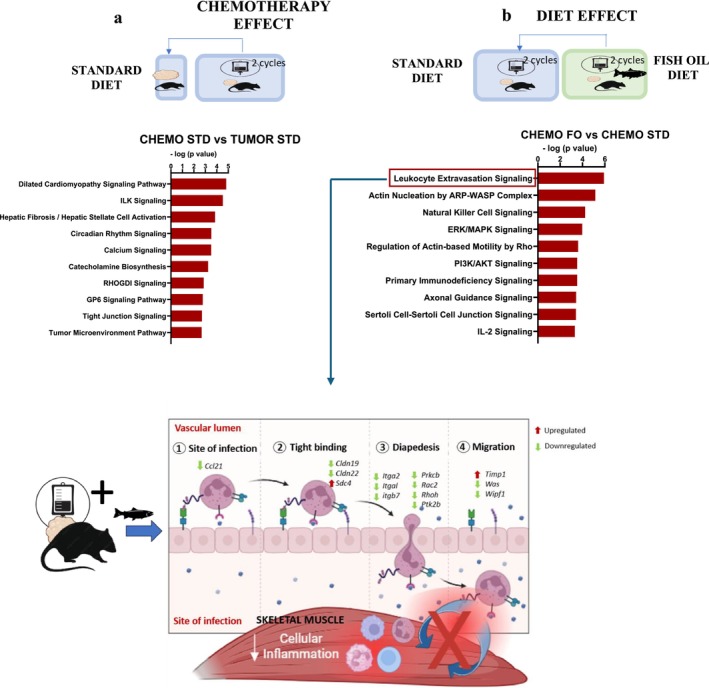
Top pathways altered by chemotherapy on a standard and fish oil diet (EPA + DHA). (a) Chemotherapy effect: top 10 canonical pathways in the CHEMO STD versus TUMOUR STD, (b) diet effect: top 10 canonical pathways in the CHEMO FO versus CHEMO STD and (c) genes in the leukocyte extravasation pathway mostly decreased by CHEMO FO. *Ccl21*—chemokine (C‐C motif) ligand 21, CHEMO—chemotherapy, CHEMO FO—tumour‐bearing animals that received chemotherapy, consumed fish oil diet, started the same day as chemotherapy (*n* = 8), CHEMO STD—tumour‐bearing animals that received chemotherapy, consumed control diet (*n* = 7), *Cldn19*—claudin 19, *Cldn22*—claudin 22, FO—fish oil, ILK—integrin‐linked kinase, *Itga2*—integrin alpha 2, *Itgal*—integrin alpha L, *Itgb7*—integrin subunit beta 7, *Prkcb*—protein kinase C beta type, *Ptk2b*—protein tyrosine kinase 2 beta, *Rac2*—Ras‐related C3 botulinum toxin substrate 2, *Rhoh*—Ras homologue family member H, *Sdc4*—syndecan‐4, STD—standard diet, *Timp1*, metallopeptidase inhibitor 1, TUMOUR STD—tumour‐bearing animals that did not receive chemotherapy, consumed control diet (*n* = 5), *Was*—WASP actin nucleation promoting factor, *Wipf1*—WAS/WASL‐interacting protein family member 1.

Rats that received chemotherapy on a standard diet exhibited significant transcriptomic changes and functional annotations associated with morphology and development, including proliferation of muscle cells (*p* < 10^−7^) and connective tissue disorder (*p* < 10^−6^) (Table 1). Myosin IIIB (*Myo3b*) was downregulated 4.16‐fold (log_2_ FC = −4.16) after two cycles of chemotherapy. Top significant canonical pathways included dilated cardiomyopathy signalling (−log*p* value = 4.82), integrin‐linked kinase (ILK) signalling (−log*p* value = 4.53), circadian rhythm signalling and calcium signalling (Figure 2a). ILK signalling is known for its role in enhancing myotendinous junction stability and alleviating muscle stress‐induced damage. The genes involved in ILK signalling, including myosin genes (*Myh11*, *Myh6*, *Myh7* and *Myh9*), were downregulated, suggesting the negative impact of chemotherapy on tight junction stability in rats on a standard diet. The Glycoprotein VI Platelet (GP6) pathway, representing the primary signalling pathway for collagen within the extracellular matrix, exhibited lower expression of genes such as collagen type 1 alpha 1 chain (*Col12a1*), *Col22a1*, *Col27a1* and *Col7a1*, suggesting chemotherapy‐induced alterations to the muscle extracellular matrix. Key genes involved in circadian rhythm regulation, such as Basic Helix–Loop–Helix Family Member E40 (*Bhlhe40*), nuclear receptor subfamily 1 group D member 1 (*Nr1d1*) and Period Circadian Regulator 3 (*Per3*), were decreased following chemotherapy administration.

#### 3.1.4 Chemotherapy Inhibited Muscle Growth and Repair Upstream Regulators

TGFβ family, as an upstream regulator involved in tissue repair mechanisms, was predicted to be inhibited (*z* score = −3.25, *p* value of overlap = 5.21E‐08). Myocardin (MYOCD; *z* score = −2.93, *p* value of overlap = 5.86E‐8) and SMAD3 (*z* score = −2.53, *p* value of overlap = 4.61E‐07) were predicted to be inhibited, indicating potential disruptions in muscle development and signalling pathways. PAX3‐FOXO1 (*z* score = 2.45), a fusion transcription factor linked to abnormal muscle cell growth, was predicted to be activated. Upstream regulators associated with muscle development and metabolism, including GATA6 (*z* score = −2.58), a transcription factor linked to muscle differentiation and MAPK1 (*z* score = −2.22), a kinase involved in muscle growth and repair, were predicted to be inhibited.

### 3.2 Transcriptomic Impact of Diet: A Comparison of Muscle Transcriptome Between Tumour‐Bearing Animals Receiving Chemotherapy and Provided a Standard Diet or a Fish Oil Diet

To evaluate the diet effect, CHEMO FO was compared to CHEMO STD. In the diet comparison, that is, CHEMO FO versus CHEMO STD, there were 274 DEGs, 200 genes (73%) were upregulated and 27% downregulated (Figure 1b). The full list of the differentially expressed genes is shown in Table S5. The Venn diagram in Figure 1c illustrates both the unique and overlapping DEG across both comparisons, revealing 40 DEGs (7.9%) that are common to both, as shown in Figure 1d. These overlapping genes reveal an opposite effect evoked by CHEMO STD versus TUM STD and CHEMO FO versus CHEMO STD in a variety of biological and physiological processes, including immune system, chemotaxis and cellular response to reactive oxygen species. We also observed five overlapping genes in the (CHEMO STD vs. TUM STD) versus (CHEMO FO vs. CHEMO STD) groups (shaded cells in Table 3). Reciprocal changes in a subset of genes support the premise for muscle homeostasis through gene expression changes in treatment groups.

**TABLE 3:** Diet effect: Top 15 highest and lowest differentially expressed genes altered by diet during chemotherapy (CHEMO FO vs. CHEMO STD).

Gene	Description	log_2_fold change	*p*
Upregulated			
*Pla2g2a*	Phospholipase A2 group IIA	5.35	8.73E‐04
*Coch*	Cochlin	3.75	9.73E‐03
*Rd3l*	RD3 like	3.37	2.39E‐03
*Kif20a*	Kinesin family member 20A	2.98	2.47E‐02
*Serpine1*	Serpin family E member 1	2.82	1.39E‐02
*Nr4a3*	Nuclear receptor subfamily 4, group A, member 3	2.71	6.22E‐03
*Cdkn1a*	Cyclin‐dependent kinase inhibitor 1A	2.63	3.58E‐03
*Selp*	Selectin P	2.60	4.18E‐03
*Myo3b*	Myosin IIIB	2.47	3.63E‐02
*Apold1*	Apolipoprotein L domain containing 1	2.47	6.16E‐04
*Fos*	Fos proto‐oncogene, AP‐1 transcription factor subunit	2.40	2.18E‐07
*Greb1*	Growth regulating oestrogen receptor binding 1	2.37	2.51E‐02
*Rrad*	RRAD, Ras‐related glycolysis inhibitor and calcium channel regulator	2.34	1.20E‐03
*Maff*	MAF bZIP transcription factor F	2.32	1.21E‐02
*Ampd3*	Adenosine monophosphate deaminase 3	2.28	2.77E‐04
Downregulated			
*Lck*	LCK proto‐oncogene, Src family tyrosine kinase	−2.87	4.55E‐02
*Ugt8*	UDP glycosyltransferase 8	−3.03	1.64E‐02
*Kif19*	Kinesin family member 19	−3.05	3.70E‐03
*Fcrl2*	Fc receptor‐like 2	−3.13	3.38E‐03
*Napsa*	Napsin A aspartic peptidase	−3.16	1.54E‐02
*Vat1l*	Vesicle amine transport 1‐like	−3.24	4.23E‐02
*Slc27a6*	Solute carrier family 27 member 6	−3.32	6.19E‐03
*Pmp2*	Peripheral myelin protein 2	−3.36	1.98E‐02
*H1f10*	H1.10 linker histone	−3.52	1.18E‐03
*Ak5*	Adenylate kinase 5	−4.17	6.08E‐03
*Dusp15*	Dual specificity phosphatase 15	−4.20	3.00E‐03
*Cldn19*	Claudin 19	−4.25	1.02E‐03
*Bcas1*	Brain enriched myelin associated protein 1	−4.78	1.42E‐03
*Ptprcap*	Protein tyrosine phosphatase, receptor type, C‐associated protein	−4.84	2.47E‐03
*Cd22*	CD22 molecule	−4.97	6.62E‐05

*Note:* Tumour‐bearing animals that received chemotherapy on a standard diet were compared to animals that received chemotherapy on a fish oil diet. Positive log_2_fold changes are upregulated genes, while negative log_2_fold changes are downregulated genes. Shaded rows are those genes that are overlapping between the chemotherapy effect and the diet effect ([CHEMO STD vs. TUM STD] and [CHEMO FO vs. CHEMO STD]). *p* value < 0.05 was considered significant.

Abbreviations: CHEMO—chemotherapy, FO—fish oil, STD—standard diet.

#### 3.2.1 EPA + DHA Modulated Immune Functions Through Inhibition of Inflammatory Pathways

CHEMO FO group elicited specific immune‐modulatory effects that were not observed with CHEMO STD. These effects included significant alterations in the quantity and activation of T lymphocytes (*p* < 10^−13^), apoptosis of mononuclear leukocytes and the quantity of granulocytes (*p* < 10^−7^), CD4 + T lymphocytes and myeloid cells (Table 1). Overall, these findings highlight the specific physiological effects of chemotherapy and the distinct roles of EPA + DHA on immune responses in a tumour‐bearing model. The full list of diseases and biological functions is shown in Table S6. Leukocyte extravasation pathway was the top enriched canonical pathway (−log*p* value = 5.92) (Figure 2b). Differential gene analysis revealed 97 canonical pathways exhibiting differential enrichment, −log(*p* value) > −log 1.3 (or *p* value < 0.05) (Table S7). Key genes crucial for various stages of leukocyte extravasation were decreased in the CHEMO FO group (Figure 2c). Leukocyte extravasation is a pivotal initiator of inflammatory processes [16]. Provision of fish oil to rats given chemotherapy led to decreased gene expression of C‐C motif chemokine ligand 21 (*Ccl21*), claudin 19 (*Cldn19*), claudin 22 (*Cldn22*), integrin alpha‐2 (*Itga2*), integrin alpha L (*Itgal*) and integrin beta‐7 (*Itgb7*), which are involved in leukocyte chemoattraction, adhesion to endothelial cells, migration and diapedesis. In summary, the provision of dietary EPA + DHA exerted multiple effects concurrently on genes involved in the leukocyte extravasation pathway.

In the CHEMO FO versus CHEMO STD comparison, activation of upstream regulators involved in immune response and inflammation was predicted (Table S8). Methylprednisolone, a corticosteroid known for its anti‐inflammatory effects on muscle tissue, was predicted to be activated. Additionally, STAT6 (*z* score = 2.36), a transcription factor that plays a critical role in cytokine signalling and modulating muscle immune responses, was also predicted to be activated.

#### 3.2.2 Dietary EPA + DHA Upregulated Membrane Lipid Remodelling Gene

Dietary intervention with EPA + DHA (CHEMO FO) significantly influenced metabolic gene expression. With the provision of dietary EPA + DHA, the most DE gene was phospholipase A2 group IIA (*Pla2g2a*) (log_2_ FC = 5.35, Table 3), which is involved in membrane lipid remodelling and inflammatory lipid mediator synthesis. This suggests enhanced phospholipid turnover and a potential adaptive response to chemotherapy‐induced membrane stress.

#### 3.2.3 Key Genes Involved in Leukocyte Migration and Cytoskeletal Dynamics Were Suppressed by Dietary EPA + DHA

CHEMO FO had reduced expression of protein kinase C beta (*Prkcb*), Ras‐related C3 botulinum toxin substrate 2 (*Rac2*), Rho‐related GTP‐binding protein RhoH (*Rhoh*), syndecan‐4 (*Sdc4*), protein tyrosine kinase 2 beta (*Ptk2b*), Wiskott–Aldrich syndrome protein (*Was*) and WASP‐interacting protein (*Wipf1*), affecting downstream signalling pathways and cytoskeletal rearrangement necessary for leukocyte migration. Furthermore, the CHEMO FO group had reduced expression of genes in other pathways, including actin nucleation by ARP‐WASP complex, natural killer (NK) cell signalling and regulation of actin‐based motility by Rho. SMAD4 (*z* score = 2.21), a central mediator of the TGFβ signalling pathway that influences muscle repair and fibrosis, was predicted to be activated in the CHEMO FO group, indicating potential enhancement of tissue remodelling and healing processes.

#### 3.2.4 EPA + DHA Inhibited Regulators of Stress Response

Upstream regulators linked to muscle proteostasis and cellular stress responses were predicted to be inhibited. These included daidzein (*z* score = −2.65), a phytoestrogen with possible muscle‐protective properties, and heat shock protein 90 (HSP90; *z* score = −2.20), a molecular chaperone essential for maintaining muscle protein homeostasis and managing cellular stress.

## 4 Discussion

We conducted an unbiased mRNA expression analysis using NGS to identify transcriptomic alterations in gene expression patterns and employed pathway analysis tools to decipher biological functions associated with muscle morphology, development and cell death as well as unique inflammatory signatures that occur after provision of CPT‐11 and 5‐FU. Dietary EPA + DHA largely protected the muscle from inflammatory responses, even when it was provided concurrently with the first injection of chemotherapy, during a time when food intake was significantly reduced. This immediate effect of diet may point to a clinically applicable nutritional strategy to mitigate chemotherapy toxicity effects at the muscle level.

Detrimental effects of chemotherapy on skeletal muscle in both human and experimental studies have been reported [1, 3]. Here, comparative pathway analysis between tumour‐bearing and those receiving chemotherapy highlighted the substantial influence of chemotherapy on signal transduction and cellular process es. The observed downregulation of several genes within the ILK signalling pathway indicates a potential adverse impact of chemotherapy on the stability of tight junctions and as detriments to muscle structural integrity. ILK signalling plays a critical role in fortifying myotendinous junctions, mitigating muscle stress‐related damage and anabolic pathways [17]. The decreased expression of genes associated with GP6 signalling, a primary receptor for collagen within the extracellular matrix [18], implies the potential of chemotherapy‐induced fibrosis in muscle.

Chemotherapy resulted in the upregulation of several genes involved in immune responses and cellular stress. Components of the classical complement pathway, such as *C1qb* and *C1qc*, along with *C3*, were increased following chemotherapy. Higher gene expression of *C3*, a central factor in complement system activation, may potentially mediate inflammation, leukocyte adhesion and migration, all of which are relevant to muscle responses and align with previous studies that reported activation of the complement system in cancer [19, 20, 21]. *Cd209f*, an immune receptor pivotal for initiating innate immunity and triggering adaptive immune responses [22], exhibited the highest upregulation by chemotherapy, suggesting increased immune activity in muscle, possibly in response to the presence of tumours [23] and chemotherapy‐induced stress. Further evidence for a direct impact of chemotherapy on inducing stress is provided by the differential expression of growth arrest and DNA damage‐inducible gamma (*Gadd45g*), known for its involvement in DNA damage and stress responses [24, 25]. In summary, chemotherapy (CPT‐11 + 5‐FU) exerts a multifaceted influence on signal transduction and cellular processes, impacting pathways crucial for muscle integrity, extracellular matrix composition, circadian rhythm signalling and inflammatory response.

Dietary EPA + DHA mitigated immune responses and inflammation in muscle, which were shown at the biological functions and pathway levels and align with other studies that have reported the anti‐inflammatory effects of dietary EPA + DHA in the muscle at the transcriptomic level [26, 27]. One of the notable effects of dietary EPA + DHA was the suppression of pathways associated with leukocyte extravasation signalling. Leukocyte extravasation is a critical step in the inflammatory response, involving the migration of immune cells from the bloodstream into tissues [28, 29]. The attenuation of leukocyte extravasation signalling suggests that dietary EPA + DHA can potentially mitigate the entry of immune cells into skeletal muscle. Several genes associated with NK cell activation and function were downregulated in the muscle of rats provided the fish oil diet. NK cells are important effectors of innate immunity, playing a role in immune surveillance and defence against infected or transformed cells [30, 31] by releasing cytokines and chemokines, connecting innate immunity to adaptive immunity [32]. Modulating NK cell signalling may impact the immune response within skeletal muscle. *Pla2g2a* expression was higher following dietary EPA + DHA. Little is known about the role of *Pla2g2a* in skeletal muscle in cancer; however, it is known to hydrolyse fatty acids from phospholipids. Fatty acids released from membranes are substrates for bioactive lipid mediators and provide initiating events for intracellular cascades involved in inflammation as well as signals for anabolic responses [33, 34]. In tumours, *Pla2g2a* has been identified as an independent predictor of survival [35] that is modified by provision of a mixed oil containing fish oil in gastric cancer patients receiving chemotherapy [36].

Dietary EPA + DHA appears to exert protective effects on skeletal muscle through the modulation of upstream regulators associated with inflammation, immune response and cell survival to circumvent the impact of chemotherapy. The inhibition of pro‐inflammatory regulators like IL33, IL1B and NFKB1 suggests mitigation of inflammatory pathways implicated in muscle damage [37, 38, 39]. Additionally, the activation of signalling molecules such as TGFβ, SMAD3 and STAT6 by dietary EPA + DHA indicates potential roles in promoting tissue repair, anti‐inflammatory responses and muscle regeneration. The transcription factor STAT6 mediates direct repression of inflammatory enhancers [40]. We noted that despite a significant reduction in food intake following chemotherapy in both standard and dietary EPA + DHA groups, the short duration of feeding with a diet containing EPA + DHA, even at reduced intakes, was sufficient to induce marked transcriptomic changes that occurred following chemotherapy in this animal model (Tables S4 and S8).

A strength of our study is the translational nature of the model. This model provides a standard chemotherapy regimen to tumour‐bearing animals, unlike other models that provide chemotherapy to healthy rodents. This enables the study of the impact of chemotherapy in a clinically relevant setting while disentangling the independent impact of the tumour. The biology is complex, and in gene‐based associations, it is critical to have a robust phenotype, and our experimental design enables a systematic analysis of pathways or networks affected by tumour, chemotherapy and EPA + DHA. While the preclinical model provided valuable insights, it may not perfectly replicate the human condition. Thus, validation using human muscle biopsies is necessary to confirm these findings in human cancer patients.

Benefits of n‐3 fatty acids have been reported to be beneficial for muscle in several disease states, including cancer [41], but few have been conducted in the context of chemotherapy [3]. The biological underpinnings of these benefits remain elusive and require experimental models to identify mechanisms. Those that do exist are primarily derived from experimental models of limited clinical relevance (i.e., providing chemotherapy to healthy animals) and provide single agents rather than combinations as is typically applied clinically. The new clinical relevance also pertains to the fish oil being initiated at the same time as chemotherapy was delivered, suggesting early, impactful effects on muscle processes. Given the marked losses of muscle that occur during a course of chemotherapy, preventing early signals and processes would be expected to have a meaningful impact on mitigating myotoxicities that endure throughout the treatment trajectory. Readily available concentrated formulations to rapidly increase EPA and DHA levels in muscle could help mitigate muscle loss during chemotherapy by attenuating inflammatory processes. However, well‐designed, sufficiently powered randomized controlled trials are needed to establish optimal dosing, duration and patient outcomes to inform clinical guidelines.

Relatively little is known about the link between systemic inflammation and local inflammation in patients with cancer and how this contributes to the disruption in muscle. Our prior work shows that systemic inflammation in humans with cancer, assessed by circulating C‐reactive protein (CRP) levels, is related to the presence of leukocytes within muscle [42]. By subjecting human biopsy material to gene microarray, we discovered that extravasation of leukocytes is a major pathway that correlates with plasma CRP, a measure of systemic inflammation that also relates to poor outcomes in surgical patients [43]. It is this pathway that appears to be modified by providing fish oil in the diet. Elevated numbers of CD8 T cells have been reported in patients with higher muscle mass [44]. However, immune cell functions within the muscle as a contributor to muscle depletion in the tumour‐bearing state have not been characterized. Therefore, our findings show a mitigation of chemotherapy's impact on muscle in this translational model and provide further evidence that inflammatory events are modified by a diet containing EPA and DHA, even at low intakes.

We have comprehensively characterized the transcriptomic changes within the muscle of rats bearing tumour + chemotherapy (with or without the provision of EPA + DHA) to gain insights into biological pathways in a highly controlled experimental setting. This unbiased approach has allowed us to study the mRNA profiles utilizing the state‐of‐the‐art NGS technology without restricting ourselves to a candidate gene quantification methodology. The novel findings include downregulation of genes involved in leukocyte extravasation when fish oil is provided in the diet. This is encouraging given that there are currently limited therapeutics for myotoxicity induced by systemic therapies. This research opens avenues for future hypothesis testing studies targeting specific biological processes.

## Conflicts of Interest

The authors declare no conflicts of interest.

## Supporting information


**Table S1:** Differentially expressed upregulated and downregulated genes (effect of chemo vs. tumour bearing, both on standard diet).
**Table S2:** Diseases and biological functions (chemo vs. tumour, both on a standard diet).
**Table S3:** Top significant canonical pathways identified (chemo vs. tumour, both on a standard diet).
**Table S4:** Upstream regulators sorted by *p* value (chemo vs. tumour bearing, both on a standard diet).
**Table S5:** Differentially expressed upregulated and downregulated genes (chemo with fish oil vs. chemo with standard diet).
**Table S6:** Diseases and biological functions (chemo with fish oil vs. chemo with standard diet).
**Table S7:** Top significant canonical pathways (chemo with fish oil vs. chemo with standard diet).
**Table S8:** Top 200 upstream regulators sorted by *p* value (chemo with fish oil vs. chemo with standard diet).

## Data Availability

All data can be found in the manuscript or the Supporting Information. Requests for materials or correspondence should be directed to the corresponding author.
